# Enhancing Theory of Mind in Children with Autism Through Computer-Assisted Instruction and the Thought-Bubble Strategy

**DOI:** 10.3390/bs16081350

**Published:** 2026-08-06

**Authors:** Yunhuan Pu, Wei Zhao, Miao Li, Zhuo Chen

**Affiliations:** 1 Faculty of Education, Shaanxi Normal University, Xi’an 710062, China; pyh1022@163.com (Y.P.);; 2 College of Teacher Education School, Sichuan University of Arts and Science, Dazhou 635000, China; 3 Department of Curriculum & Instruction, College of Education, University of Houston, Houston, TX 77204, USA

**Keywords:** children with autism, computer-assisted instruction, theory of mind, thought-bubble strategy

## Abstract

Children with autism spectrum disorder (ASD) have significant deficits in communication, emotional recognition, perspective-taking, and social skills. Evidence from existing studies has shown that the social interaction skills of children with ASD are closely related to their theory of mind (ToM) abilities. While prior research has explored the effect of single intervention modes such as computer-assisted instruction (CAI) and the thought-bubble strategy, the efficacy of combining CAI with the thought-bubble strategy remains unclear. This study investigates the effectiveness of this multifaceted approach in fostering ToM development in children with ASD. Using a single-case design, the intervention for two children with ASD involved implementing CAI combined with the thought-bubble strategy to teach ToM skills to children with ASD, including basic beliefs, first-order beliefs and first-order false beliefs. Results indicated that both children showed an increase in their ToM abilities, evidenced by their increased tendency to use “I think/I feel” statements in everyday life, enhanced understanding of false beliefs, and a decrease in negative emotions. The thought-bubble strategy can be embedded in CAI, and the combination of these two methods facilitates the enhancement of the ToM abilities among children with ASD with good maintenance and generalization effects.

## 1. Introduction

Autism spectrum disorder is a neurodevelopmental disorder in which impaired social interaction is one of the core symptoms (ASD; *Diagnostic and Statistical Manual of Mental Disorders*, Fifth Edition, *Text Revision*; American Psychiatric Association, 2022). Specifically, individuals with ASD exhibit significant deficits in theory of mind (ToM): the ability to understand others’ mental states (Baron-Cohen et al., 1985). More precisely, children with ASD have difficulties in identifying and understanding others’ emotions and feelings, which impairs their social adaptation. Current interventions designed to improve ToM in children with ASD include (a) computer-assisted instruction (CAI), which creates a structured digital learning environment to teach children with ASD (Balderaz & Mehta, 2016), and (b) the thought-bubble strategy, which is a visual scaffolding technique that represents mental states through bubbles (Wellman et al., 2002). Although each approach has been proven effective, no study has been conducted to examine the potential influence of combining CAI with the thought-bubble strategy on the development of ToM among children with ASD, especially in intervention research involving Chinese-speaking children with ASD. Therefore, the present study employed a single-case design with a multiple-probe design across behaviors to investigate the effectiveness of CAI combined with the thought-bubble strategy in enhancing the ToM competence of Chinese-speaking children with ASD, targeting basic beliefs, first-order beliefs and first-order false beliefs as specific intervention points.

### 1.1. ToM Deficits in Children with ASD

ToM refers to the capacity to understand one’s own mental states such as emotions, beliefs, desires, and intentions, and to infer and interpret the mental states and behaviors of others. It is a fundamental prerequisite for effective social cognition and interpersonal communication (Baron-Cohen et al., 1999). Importantly, existing theoretical frameworks emphasize the intrinsic link between social cognitive abilities and language/communication capacities, suggesting that these domains are strongly interconnected in both neurotypical and atypical development (Oesch, 2024). This interconnectedness indicates that interventions designed to enhance ToM may effectively support both social understanding and communication skills. Considering that children with ASD exhibit co-occurring deficits in these areas, interventions for the development of ToM should be designed and provided. According to Steele et al. (2003), ToM development progresses through a hierarchical sequence of three stages: (a) an early stage involving desire understanding, pretend play, and the use of mental state language; (b) a foundational stage characterized by emotion understanding, knowledge access, and first-order false-belief understanding; and (c) a higher-order stage encompassing second-order false-belief understanding, strategic deception, and comprehension of nonliteral language.

Baron-Cohen et al. (1985) initially posited that deficits in social interaction observed in autism are primarily attributable to impairments in ToM. Subsequent empirical studies have demonstrated a significant correlation between ToM abilities and social functioning in children with ASD (e.g., Adibsereshki et al., 2015; Altschuler et al., 2018; Andreou & Skrimpa, 2020; Chiu et al., 2023; Mao et al., 2023; Żuromski & Pacholik-Zuromska, 2026). According to Howlin et al. (1999), typically developing children are capable of understanding contextual emotions and possessing elementary false beliefs by the age of 4 to 5 years. However, children with ASD consistently lag behind their typically developing peers in the acquisition of ToM skills (e.g., Begeer et al., 2003; C. C. Peterson et al., 2005; Loukusa et al., 2014; C. Peterson et al., 2016; Offek & Segal, 2022). Research evidence indicates that children with ASD exhibit specific deficits in core ToM components, including difficulties distinguishing appearance from reality, understanding that appearance does not necessarily reflect underlying reality (“seeing leads to knowing”), perspective-taking, and mastering first-order false beliefs (Baron-Cohen et al., 1999). Notably, even after controlling for non-verbal IQ, ToM abilities in children with ASD remain markedly inferior to those of both typically developing children and children with language impairments (Offek & Segal, 2022).

### 1.2. ToM Interventions for Children with ASD

To address these challenges, various ToM intervention strategies have been developed, including systematic teaching procedures (Ma et al., 2023), thought bubbles (Wellman et al., 2002), CAI (Balderaz & Mehta, 2016), and multiple exemplars of instruction (Lovett & Rehfeldt, 2014). Among these, the thought-bubble strategy has been considered an effective strategy that can concretize abstract mental states. By visualizing thoughts in a graphic format, it reduces the cognitive load on children with ASD, who often find abstract thinking challenging (Wellman et al., 2002). Similarly, CAI is recognized as a promising approach for engaging children with ASD through structured, predictable, and multisensory presentations (e.g., visual cues and auditory feedback), which cater to their learning profiles and enhance attention (Good et al., 2024).

Given the strong theoretical foundation and empirical support for both the thought-bubble technique in demystifying mental states and CAI in addressing the learning needs of children with ASD, this study evaluates the combined contribution of these two interventions to ToM development among children with ASD.

### 1.3. The Thought-Bubble Strategy Increases ToM Abilities for Children with ASD

A thought bubble is a visual representation (i.e., typically a cloud-like image above a character’s head) that depicts internal thoughts, making individuals’ mental states observable to others (Wellman et al., 2002). Research on using thought bubbles to enhance ToM in children with ASD has evolved through two main methodological approaches, which are group experiments and single-case designs. The study by Wellman et al. (2002) demonstrated its effectiveness in teaching first-order false-belief understanding, primarily using pre–post controlled group designs. More recent research has shifted toward single-case studies, which have replicated these positive effects, including maintenance over time, even in Chinese populations (Zhang et al., 2016).

Despite these promising findings, two key limitations have been identified. First, most interventions remain paper-based, which limits the interactivity and ecological validity. Second, assessments often focus narrowly on immediate task performance and lack a comprehensive, multidimensional approach to measuring generalization across different ToM components. Therefore, while the thought-bubble strategy is validated, there are significant opportunities to digitize intervention tools and develop more robust assessment frameworks that track progress from baseline through long-term generalization.

### 1.4. CAI Increases ToM Abilities for Children with ASD

CAI has been extensively employed in interventions for children with ASD, leveraging its structured format and visual supports to capitalize on their strengths in visual processing and enhance engagement (Snyder & Huber, 2019). Systematic reviews have validated the effectiveness of CAI in improving social cognition, often demonstrating superior outcomes compared to traditional intervention methods (Hu et al., 2020; Lee et al., 2015). Nonetheless, the application of CAI to the development of ToM remains relatively limited. One significant obstacle is poor generalization; skills acquired through computerized tasks frequently fail to transfer effectively to real-world social contexts, suggesting a reliance on rote memorization rather than genuine understanding (Swettenham, 1996). Additionally, many CAI interventions tend to target isolated components of ToM, such as emotion recognition, neglecting the complex, stage-dependent progression of ToM development, which constrains their broader translational impact.

Integrating the thought-bubble strategy with CAI offers a promising avenue to enhance ToM instruction in children with ASD. The thought-bubble technique effectively externalizes internal mental states, rendering abstract concepts more concrete and accessible (Wellman et al., 2002). However, its traditional static presentation often lacks intuitiveness and necessitates extensive adult guidance, limiting scalability and engagement (Arpacık et al., 2023). CAI can directly address these limitations by providing dynamic visualizations, real-time interactive feedback, and engaging multimedia elements, thereby making mental state representations more intuitive and engaging (Stevenson et al., 2016). The synthesis of the conceptual clarity of thought bubbles with CAI’s interactive capabilities has the potential to produce scalable, efficacious interventions that foster comprehensive ToM development. Such integrated approaches may offer more accessible, engaging, and effective methods to promote generalization and deepen understanding of social cognition for children with ASD.

### 1.5. The Present Study

This study utilized CAI in conjunction with the thought-bubble strategy to conduct a systematic intervention study of the ToM abilities of 6-to-8-year-old children with autism. The study employed a single-case design with a multiple-probe design across behaviors, focusing on basic beliefs, first-order beliefs, and first-order false beliefs. During the intervention, CAI provided a structured learning environment, and the thought-bubble strategy visualized the inner mental states of the characters through visualization, both of which were designed to work together to promote the development of ToM abilities in children with autism. The study comprehensively examined the immediate, maintenance, and generalization effects of the intervention through systematic assessments in the baseline, intervention, maintenance, and generalization periods, providing new empirical evidence and practical guidance for the intervention aimed at enhancing the ToM ability of children with ASD.

## 2. Method

### 2.1. Participants

Participants were children aged 6 to 8 years with a clinical diagnosis of autism, recruited via WeChat (a commonly used mobile social media app in China) through a local parents’ association in a southwestern city of China. Following a screening process, two male children met all inclusion criteria and participated in the study. The inclusion criteria were as follows: (1) a formal autism diagnosis confirmed by licensed clinicians utilizing standardized assessment instruments such as the Autism Diagnostic Observation Schedule-2 (ADOS-2) or the Autism Diagnostic Interview—Revised (ADI-R); (2) a ToM score below 35, as delineated by Feng (2001), indicating significant difficulties in mentalizing abilities; and (3) evidence of functional verbal communication, demonstrated by the child’s ability to respond appropriately to wh-questions. Informed consent was obtained from the parents of all participants.

Tu, a 7-year-and-6-month-old boy, had a Childhood Autism Rating Scale (CARS; Schopler et al., 2002) score of 32, indicating moderate autism severity. His Full-Scale IQ, assessed with the Wechsler Intelligence Scale for Children—Fourth Edition, was 75, placing him within the borderline intelligence range. Tu demonstrated strengths in object naming, arithmetic reasoning, telling time, Pinyin spelling, and word reading. His functional communication skills were evidenced by appropriate responses to wh-questions and the ability to maintain conversational topics. Additionally, he exhibited emerging social–emotional skills, including basic theory of mind awareness and reciprocal interactions.

Xuan, aged 6 years and 8 months, was a kindergarten attendee. He scored 33 on the CARS, consistent with moderate autism severity, and achieved a Full-Scale IQ of 79 on the WISC-IV. Assessments using the Developmental Native Behavioral Rating System (Feng et al., 2015) revealed foundational cognitive abilities, such as object naming and sight-word reading, alongside emerging social–emotional skills, including desire-based emotion recognition, symbolic play, and early ToM competencies.

### 2.2. Setting and Materials

#### 2.2.1. Setting

The study was conducted in a special education training room at a university in southwest China. Each room was equipped with a child-sized table, chairs, and a video camera. During the study, the teacher and child sat facing each other across the table.

#### 2.2.2. Materials

This study employed three assessment instruments: the Test of Theory of Mind (TToM), the Developmental Native Behavioral Rating System for Children with ASD, and the Teaching Objectives Test.

**The Developmental Native Behavior Rating System for Children with ASD (Feng et al., 2015).** The Developmental Native Behavioral Rating System for Children with ASD was utilized to assess participants’ performance across cognitive, communicative, and socio-emotional domains, with particular emphasis on ToM-related competencies within the socio-emotional dimension. The scale encompasses five primary domains: cognitive ability, communication ability, social–emotional ability, motor ability, and adaptive ability. The social–emotional domain consists of seven subcomponents: non-verbal social emotions, emotional behaviors, play behaviors, ToM abilities, social skills in interpersonal interactions, social responsibility, and problem-solving. Within this domain, the ToM component comprises five sections: basic intentions (2 items), basic beliefs (3 items), first-order beliefs (1 item), first-order false beliefs (1 item), and second-order false beliefs (1 item). These were designed based on children’s developmental milestones, with each item scored on a three-point scale: 0 points for an incorrect or absent response, 1 point for a partially correct response, and 2 points for a fully correct response.

**The Test of Theory of Mind (Feng, 2001).** The test demonstrated a reliability coefficient ranging from 0.71 to 0.94 (*p* < 0.01). The Test of ToM was used for two primary purposes: (1) to screen participants and determine whether their ToM abilities fell below developmental expectations and (2) to provide a comprehensive evaluation of their ToM skills at pre- and post-intervention stages, facilitating the measurement of significant changes over time. The TToM comprises 40 items derived from eight real-life vignettes, with a total possible score of 43 points. These items cover various aspects of ToM, including situation-based emotions (8 items; total score: 8), desire-based emotions (8 items; total score: 8), basic beliefs (6 items; total score: 6), first-order false beliefs (4 items; total score: 6), second-order false beliefs (2 items; total score: 3), and factual, recollective, or implicit questions (12 items; total score: 12). The raw scores are expressed as percentages of the maximum possible scores, facilitating comparison across assessments. The same set of items was administered during pre- and post-intervention sessions, enabling a quantitative analysis of changes in children’s ToM abilities. Results are typically represented graphically to visually depict developmental progress attributable to the intervention.

**Teaching Objectives Test.** This study employed a self-developed “Teaching Objectives Test” to evaluate three targeted behaviors: the expression of “I feel”, first-order beliefs, and first-order false beliefs. The test was constructed in accordance with the operational definitions and evaluation framework for ToM as outlined in the Developmental and Behavioral Rating System for Children with Autism (Feng et al., 2015). The Teaching Objectives Test was administered at multiple points throughout the baseline, intervention, and maintenance phases. It employed parallel forms of test items to reliably measure participants’ ToM abilities repeatedly, with scoring rates serving as quantitative indicators to monitor the acquisition and consolidation of targeted instructional objectives.

The assessment consisted of five parallel, scenario-based stories for each target behavior (Wellman et al., 2002). These stories maintained a consistent narrative structure and comparable cognitive demands to ensure measurement reliability, while surface details (e.g., characters, objects, and locations) were varied to control for practice effects and contextual biases. Each story was followed by core questions directly assessing the corresponding ToM construct (Table S1). For example, a scenario illustrating first-order false beliefs was presented as follows: “Xiao Ming puts his chocolate in the drawer, then leaves. While he is away, his mother moves the chocolate to the cabinet. Later, Xiao Ming returns and wants to eat his chocolate.” Subsequent questions included: (1) behavioral prediction: “Where will Xiao Ming look for the chocolate first?” (correct answer: the drawer) and (2) justification: “Why?” (correct answer: e.g., “because he thinks it is still in the drawer” or “he didn’t see it being moved”).

A standardized scoring protocol was employed. For the “I feel” expression, a maximum of two points was awarded per scenario, with one point for using a self-referential phrase (e.g., “I think” or “I feel”) and one point for providing a contextually appropriate description. Responses to first-order beliefs and false beliefs were scored out of two points per scenario, with one point awarded for correct answers to each of the two questions. The total score for each behavioral target across five scenarios thus ranged from 0 to 10 points. All responses were independently evaluated by trained researchers using predefined rubrics to ensure scoring objectivity.

### 2.3. Experimental Design

This study employed a multiple-probe design within a single-subject experimental framework to investigate behavioral outcomes. The considerable heterogeneity characteristic of children with ASD poses challenges in recruiting a large sample with comparable abilities across multiple domains. Consequently, a single-subject approach allows for detailed examination of intervention effects with a limited number of cases. The multiple-probe design, a variation in the multiple baseline methodology, minimizes the need for extensive repeated measurements during the baseline phase. This approach effectively addresses two key limitations: it accounts for intervention goals that are inherently non-reversible and reduces the demand for numerous baseline assessments that may induce fatigue or aversion in participants. In the current study, a multi-probe design was implemented across three target behaviors: expression of “I feel” related to basic beliefs, first-order beliefs, and first-order false beliefs.

#### 2.3.1. Independent Variables

The independent variables in this study consisted of CAI integrated with the thought-bubble strategy. Specifically, CAI involved presenting visual representations of the instructional objectives, with thought bubbles serving as prompts to facilitate responses. The researcher designed electronic images aligned with the teaching goals, incorporated these visuals into PowerPoint presentations, and added subtitles and recorded voice-overs to enhance engagement and clarity. During the intervention, thought bubbles were displayed as response prompts directly on the computer screen to support participants’ comprehension and active participation.

#### 2.3.2. Dependent Variables

Pre-test assessments confirmed a global ToM impairment (Test of Theory of Mind scores < 35). A fine-grained analysis using the Developmental Native Behavior Rating System for Children with ASD revealed intact foundational skills (e.g., basic intentions; seeing leads to knowing) but consistent failures on subsequent, more advanced ToM competencies (i.e., basic beliefs, first-order beliefs, and first-order false beliefs). This profile suggests a developmental plateau at the transition to advanced ToM reasoning. To address this gap and promote progression along the ToM hierarchy, the intervention targets (a) basic beliefs (indexed by ‘I think/I feel’ statements), (b) first-order beliefs (representations of another’s belief state), and (c) first-order false beliefs (representations of beliefs that contradict reality). These targets were selected because they build directly on intact foundational skills and correspond to the identified locus of difficulty for both participants. The operational definitions of the independent variables were provided in Table 1.

### 2.4. Procedures

#### 2.4.1. Baseline

The researcher verbally articulated the stimulus stem pertinent to the target behavior and systematically recorded the children’s responses. Each child was allotted a maximum of three seconds to respond to each question, and the researcher provided neutral feedback indicating the correctness of the response. Neutral feedback was defined as evaluative language focused solely on the child’s effort or response, such as “thoughtfully”, and was intended to minimize influence on the child’s motivation or emotional state. To preserve the internal validity and stability of the baseline data, positive reinforcement or corrective feedback (e.g., praise or expressions of disappointment) were deliberately avoided.

The study adhered to established protocols for single-case experimental designs, particularly regarding the criteria for phase changes. Specifically, the intervention phase for the “basic beliefs” component was initiated only after the baseline data demonstrated a stable pattern, defined according to standard stability criteria. The first-order belief component was introduced into the intervention phase once the baseline data indicated stability and the criterion of 100% correct responses was consistently met over two consecutive days. Subsequently, the first-order false-belief component was integrated into the intervention phase following the same stability and accuracy criteria as the first-order beliefs.

#### 2.4.2. Intervention

The intervention employed a structured, CAI protocol integrated with a visual thought-bubble strategy to target three developmental targets: “I feel/I think”, “first-order belief” and “first-order false beliefs”. The teaching was grounded in Discrete Trial Training (DTT), with each session comprising discrete trials that followed a cyclical sequence: stimulus presentation, prompt delivery, child’s response, feedback, and an inter-trial interval.

Each session began with a pre-test trial without prompts to assess the baseline, followed by neutral feedback (e.g., “OK”). Subsequently, five consecutive teaching trials were implemented using systematically applied prompt strategies: “P” for prompting or “+” for independent responding. Prompting strategies were dynamically adjusted via a fading procedure. The “P” strategy involved immediately presenting a prompt screen with thought bubbles after the instruction, typically during initial acquisition. In contrast, the “+” strategy required an independent response within a three-second delay. To foster independence, a three-second delay was introduced before the “P” prompt from the fourth or fifth trial onward. The post-test outcome determined the subsequent session’s strategy: correct independent responses led to the “+” strategy, while errors reinstated the “P” strategy.

**Figure 1:**
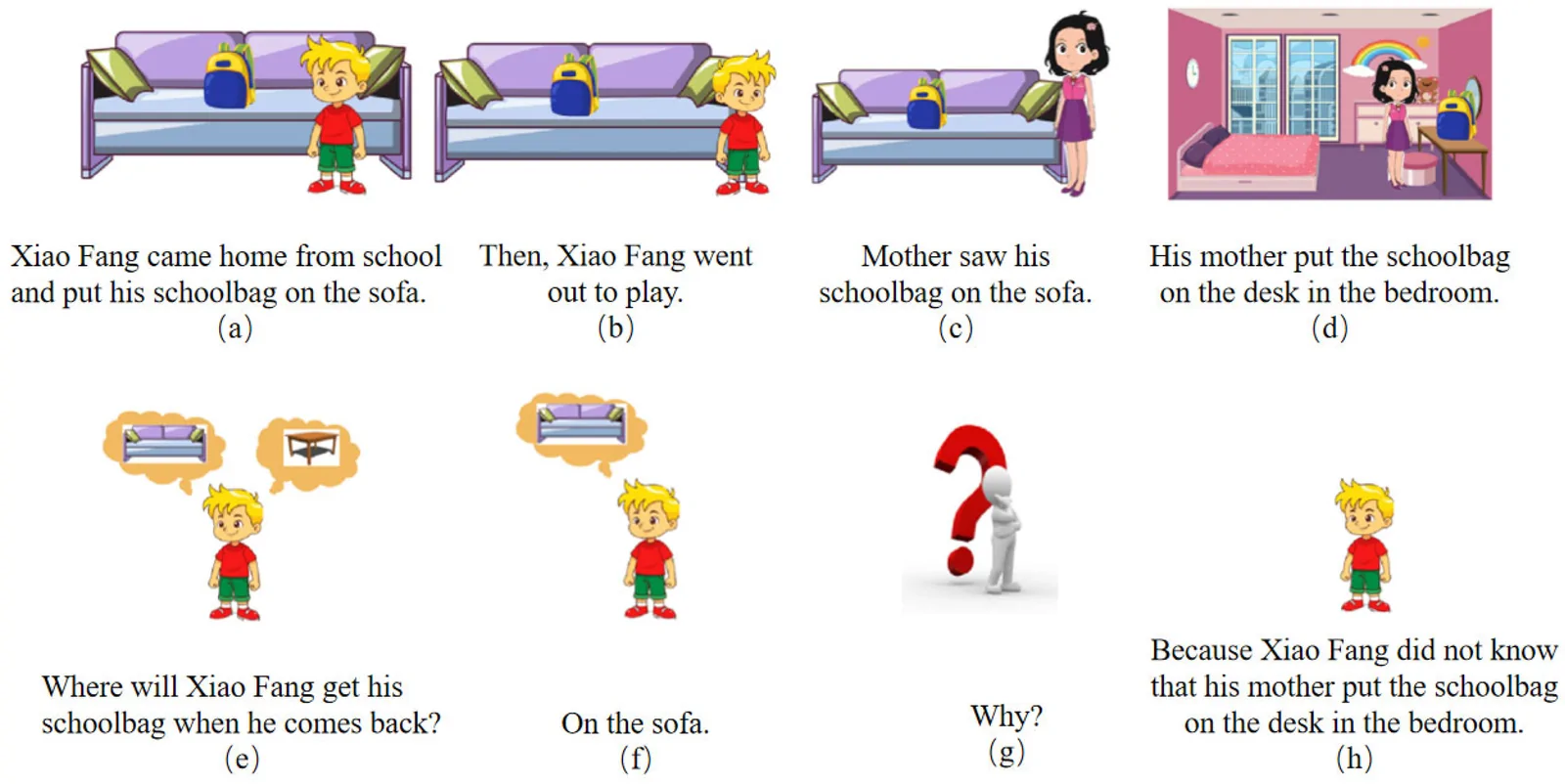
Teaching materials of the first-order false belief (original work by the authors).

Each teaching trial was structured into five steps. Stimulus Presentation: The computer displayed dynamic visual stimuli (a–e), accompanied by oral narration and the target question from the researcher (Figure 1 for the teaching materials of the first-order false belief). Prompt Delivery: Depending on the assigned strategy, a prompt screen (f) containing the thought bubble was presented either immediately or after a delay. Child’s Response: The child provided a verbal response. Feedback and Data Recording: Correct prompted responses (“p”) and correct independent responses (“+”) received descriptive praise. Incorrect or absent responses (“−”) were followed by an error correction procedure involving re-instruction and prompting. Inter-trial Interval: A 3–5 s pause was implemented, during which the researcher occasionally inserted maintenance tasks to sustain motivation.

Mastery was defined as the child achieving independent correct responses (“+”) across all five teaching trials for two consecutive sessions, upon which the next exemplar was introduced.

### 2.5. Follow-Up

The researcher refrained from engaging in any teaching or prompting activities during these phases; the procedures closely resembled those used during the baseline condition. Specifically, the researcher dictated the test stimuli and posed questions without providing cues or feedback, thereby observing the child’s spontaneous responses. Response data were systematically recorded during these assessments. Maintenance data were collected at three time points—one week, two weeks, and one month post-intervention—to evaluate the durability and retention of the learned behaviors. In this study, a single session of contextual generalization was conducted during the intervention phase and once during the maintenance phase. The researcher collaborated with the children’s parents to develop real-life scenarios that could be simulated within the teaching context, integrating the instructional objectives with the children’s daily routines and learning environments.

### 2.6. Statistical Analyses

The results of the single-case experimental design were primarily analyzed through visual analysis, which incorporated both within-phase and between-phase dimensions. Within-phase analysis included measures such as phase mean, trend slope, and trend stability. Between-phase analysis assessed changes in level, trend stability, and effect size using Tau-U. In this study, the immediate effects of the CAI combined with the thought-bubble strategy on children with ASD’s ToM abilities were evaluated based on mean level changes and Tau-U values across phases. Tau-U is an effect size metric that quantifies intervention effectiveness by measuring the degree of data overlap between baseline and intervention phases (Parker et al., 2011).

#### 2.6.1. Interobserver Agreement and Fidelity of Implementation

Interobserver agreement (IOA) was assessed by a second special education professional who independently scored 30% of recorded sessions across all phases and participants. Prior to formal scoring, both observers were trained on the scoring criteria to achieve at least 80% agreement. IOA was calculated for each target and phase. The mean IOA was 94% (range: 90–100%) for Tu and 96% (range: 90–100%) for Xuan.

Procedural fidelity was evaluated by an independent expert who reviewed 30% of instructional videos using a procedural checklist. Implementation fidelity was 100% for Tu and 98% for Xuan.

#### 2.6.2. Social Validity Questionnaire

To assess the appropriateness of the selected teaching objectives, the rationality of the instructional process, and the overall effectiveness of the intervention, a social validity scale was developed and administered to parents. The questionnaire comprised ten items: the first eight were closed-ended, and the last two were open-ended. Closed-ended items were scored on a 5-point Likert scale, with 1 indicating strong disagreement and 5 indicating strong agreement.

The first three questions evaluated the appropriateness of the teaching objectives, including whether they addressed children’s developmental needs, whether they targeted skills currently being developed, and whether the children had previously received interventions related to ToM. Questions 4–7 assessed the reasonableness of the instructional process, focusing on the session length, frequency, potential to enhance children’s motivation, and the teacher’s proficiency. The final three items addressed teaching effectiveness, specifically whether there was a significant improvement in ToM abilities, whether children could generalize these skills to interpersonal interactions, and parents’ suggestions for future improvements to the CAI program combined with the thought-bubble strategy for ToM education and research.

## 3. Results

**Figure 2:**
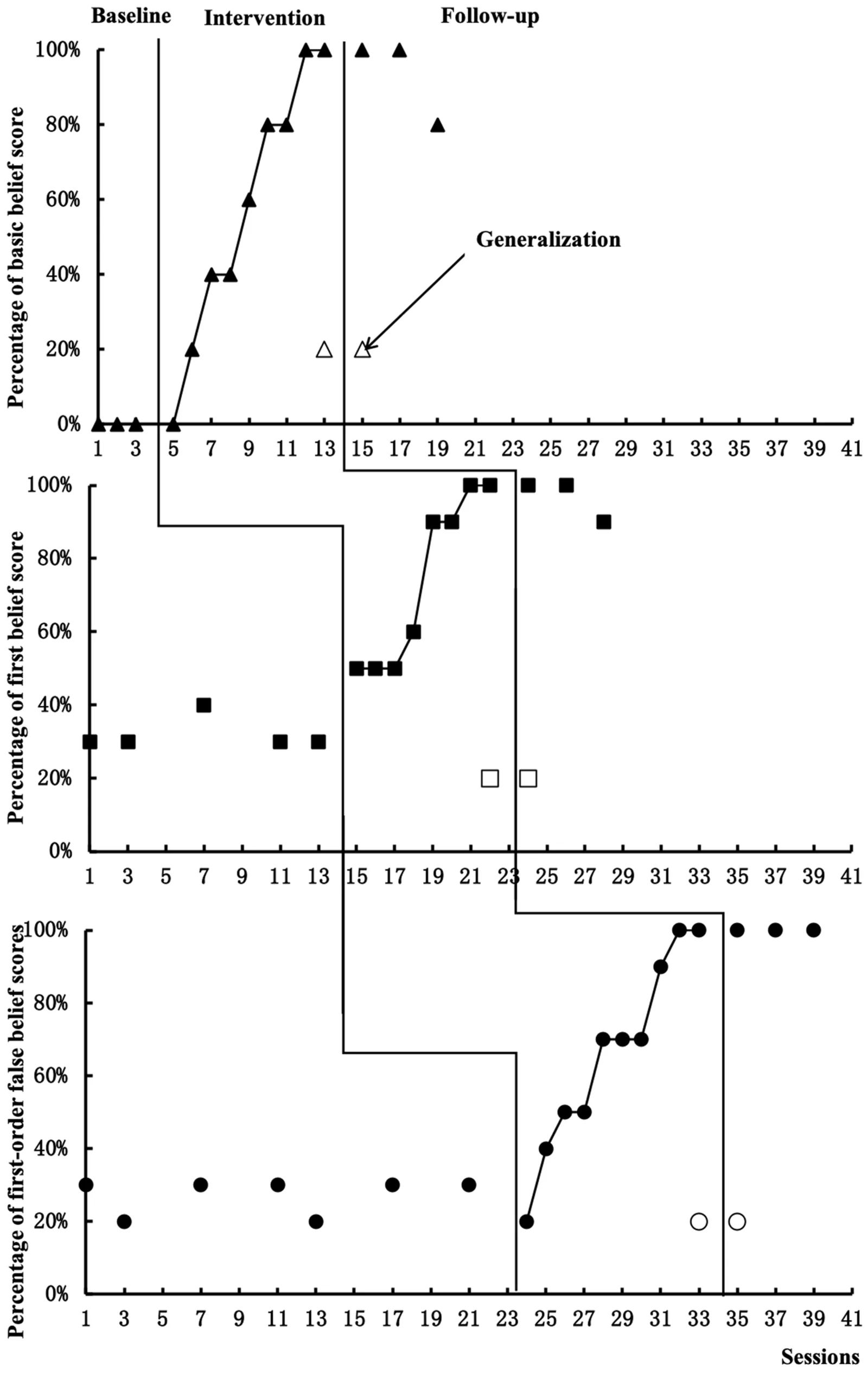
Effect diagram of theory of mind intervention for Tu.

**Figure 3:**
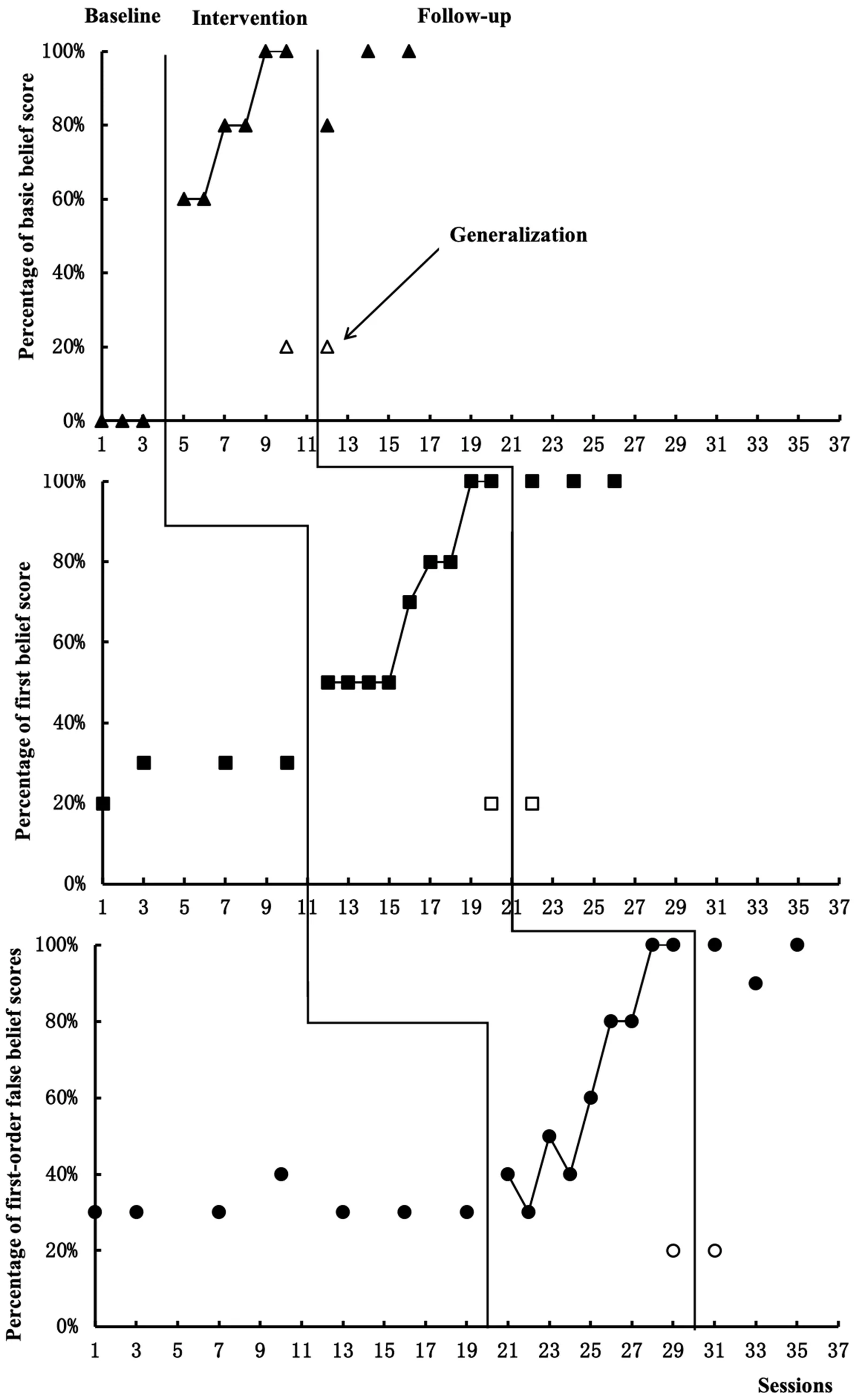
Effect diagram of theory of mind intervention for Xuan.

Figure 2 and Figure 3 show Tu and Xuan’s percentage scores of children’s basic beliefs, first-order beliefs, and first-order false beliefs before and after the intervention of the CAI combined with the thought-bubble strategy.

### 3.1. Analysis of the Results of the ToM Intervention for Two Children

#### 3.1.1. Analysis of the Results of Tu’s ToM Intervention

CAI combined with the thought-bubble strategy produced immediate, maintenance, and generalization effects on three target ToM behaviors in participant Tu. Regarding immediate effects, the “I think/feel” behavior was at 0% during baseline. Following intervention, the average level immediately rose to 57% (range 0–100%), with a clear upward trend. The Tau-U effect size was 0.89, indicating a significant effect. The “first-order belief” behavior was stable at 32% (range 30–40%) during baseline, increasing to an average of 74% (range 50–100%) post-intervention with a stable upward trend; Tau-U was 1.00, reflecting a strong and statistically significant effect. The baseline mean for “first-order false belief” was 27% (range 20–30%), which increased to 66% (range 20–100%) after intervention. Despite increased variability, the overall trend was upward, with a Tau-U of 0.83 compared to baseline. During the maintenance phase, all three behaviors were sustained at elevated levels (93%, range 80–100%; 97%, range 90–100%; and 100%) with minimal variability, indicating the effectiveness of the maintenance. Generalization was assessed via two novel contextual probes per target behavior, administered in separate sessions. Tu responded correctly on 100% of these trials, providing preliminary evidence of generalization despite the limited number of probes.

#### 3.1.2. Analysis of the Results of Xuan’s ToM Intervention

The combination of CAI with the thought-bubble strategy yielded immediate, maintenance, and generalization effects on three ToM-related target behaviors in Xuan. Immediately following the intervention, the “I think/feel” behavior remained at 0% during baseline, but increased to an average of 80% (range 60–100%), with an accelerating upward trend. The Tau-U effect size of 1.00 indicates a significant immediate effect. Similarly, the “first-order belief” behavior was stable at 28% (20–30%) during baseline, rising to an average of 70% with a stable upward trend post-intervention; Tau-U was 1.00, signifying a strong effect. The “first-order false belief” behavior showed a baseline mean of 31% (30–40%), increasing to 64% (30–100%) post-intervention, with increased variability but a clear upward trend; Tau-U was 0.84. These effect sizes confirm the immediate efficacy of the intervention. During the maintenance phase, all behavioral measures remained consistently elevated (93%, range 80–100%; 100%; and 97%, range 90–100%) with minimal variability, indicating the effectiveness of the maintenance. Generalization was assessed via single-trial probes involving new contextual tasks for each target behavior.

#### 3.1.3. Comparative Analysis of the Results of the ToM Test for Two Children

The ToM assessment focused on four objectives: situational emotions, desire-based emotions, basic beliefs, and first-order false beliefs. Both children demonstrated high competence in situational emotions, with scores of 7 and 8 out of 8, indicating alignment with the targeted teaching focus on basic beliefs. Prior to the intervention, their abilities in desire-based emotions, basic beliefs, and first-order false beliefs were limited, with the absence of corresponding bars in the graphs indicating a score of 0 for these components. Post-intervention, Tu scored 6 points in desire-based emotions, 6 in basic beliefs, and 6 in first-order false beliefs (total score of 18 across three tasks). Xuan scored 8, 6, and 6 points respectively (out of 8, 8, and 8). These results align with findings that CAI combined with the thought-bubble strategy effectively enhances ToM abilities in 6- and 8-year-old children with ASD.

### 3.2. Social Validity Analysis

At the conclusion of the study, parents completed the Social Validity Rating Scale. Their mean scores were 4.50, 4.25, and 4.50 for the appropriateness of teaching objectives, the rationality of the teaching process, and teaching effectiveness, respectively. Both parents regarded the intervention targeting ToM abilities as developmentally appropriate and necessary. They reported that the curriculum’s duration and frequency did not interfere with daily routines or other activities, and that their children were able to cooperate and participate actively.

Parents also noted that the CAI combined with the thought-bubble strategy effectively attracted their children’s attention and stimulated interest in learning, facilitating engagement in teaching activities. Regarding outcomes, parents observed functional improvements in their children’s expressive language. Specifically, in response to open-ended Question 9, both children began to use phrases such as “I think/I want” to express their thoughts, compared to earlier behaviors. For example, Tu now says, “I want to go to the park to play” or “I want to play with blocks”, whereas previously he only referenced objects or places without linking them to personal desires.

Xuan’s mother reported enhanced social skills, including better comprehension of others’ words and actions. The frequency of unexplained tantrums decreased, and he showed improved sharing behavior—initially reluctant to share toys, he gradually learned to understand that peers wanted to play with him and became more willing to share. Additionally, Xuan’s frustration and crying when items could not be found diminished; instead, he now calmly asks for help or searches methodically.

## 4. Discussion

Results from the present study demonstrate that a targeted intervention integrating CAI with the thought-bubble strategy yielded immediate, sustained, and generalized improvements in ToM skills for children with ASD. By successfully translating skills acquired through CAI into real-world social understanding, our findings address a gap in the literature concerning the generalization of taught ToM skills. The robust transfer of skills to naturalistic situations and standardized tasks was evident; participants not only completed contextual scenario tests but also answered both unexpected location and unexpected content tasks on the ToM competency test. This transfer of skills suggests that the intervention significantly impacted the underlying cognitive construct of ToM, consistent with the classical conceptualization of false beliefs (Wimmer & Perner, 1983), rather than simply facilitating rote responses.

The successful acquisition of fundamental beliefs, first-order beliefs, and first-order false beliefs aligns with prior research on the efficacy of CAI for high-functioning children with ASD (Balderaz & Mehta, 2016), which also involved participants with IQ scores above 70 and adequate verbal skills. However, our findings crucially extend this body of literature by demonstrating clear generalization to real-life scenarios. This outcome diverges from the limited generalization reported by Swettenham (1996); we attribute this discrepancy to methodological enhancements in our study, particularly the incorporation of visual scaffolding via the thought-bubble strategy, which bridges the gap between abstract mental states and their external representation. Moreover, participant characteristics such as IQ levels above 70, which is a critical cognitive parameter not reported by Swettenham, further substantiate our findings. The observed generalization is also consistent with other studies reporting successful transfers of ToM skills (Dhadwal et al., 2021).

The interrelationship between the intervention and skill acquisition is further evidenced by significant improvements in social communication. Participants increasingly utilized “I think/I want” statements to articulate their thoughts in family and structured settings, thereby reducing misunderstandings and enhancing interactions. These results corroborate a growing body of literature indicating that gains in ToM can directly improve social functioning and quality of life (Lian et al., 2025). This aligns theoretically with Oesch (2024)’s social brain framework, in which ToM acts as a bridge between interconnected social and communicative capacities, suggesting that improvements in ToM lead to broader social competence. The effectiveness, maintenance, and generalization observed can be attributed to several well-considered factors. First, the intervention was tailored to participants’ pre-test profiles, confirming adequate language skills—a known facilitator of ToM development (Kimhi, 2014). This individualized, deficit-targeted approach ensured that instruction aligned with the children’s zone of proximal development. Second, the instructional strategy itself was pivotal. The integration of CAI with the thought-bubble method created a visually engaging learning environment that likely enhanced motivation and cognitive accessibility, making abstract ToM concepts more concrete. This synergy between technological delivery and theoretically grounded visual scaffolding appears to be a key active ingredient. Finally, the deliberate use of multiple exemplars through stories and real-life scenarios aligned with instructional goals was essential for fostering generalization, directly addressing the call from Fletcher-Watson et al. (2014) for deeper exploration into generalization strategies within CAI-based ToM interventions.

### 4.1. Limitations and Recommendations for Future Research

Although this study verified the effectiveness of CAI combined with the thought-bubble strategy in intervening in the ToM abilities of children with ASD and had an excellent generalization effect, several limitations deserve mentioning.

First, this study had a small sample size, with only two participants, both of whom had mild-to-moderate ASD. Therefore, this study serves as a pilot study in terms of sample size. Regarding intervention effectiveness, this research explored only the effects of combining the CAI with the thought-bubble strategy for children with mild-to-moderate ASD and did not address its effects on children with severe ASD. Future studies should consider increasing the sample size and including participants with severe ASD to investigate the effectiveness of this intervention method.

Second, Baron-Cohen et al. (1985) identified ToM deficits as the primary cause of social interaction challenges in children with autism, as they struggle to infer others’ thoughts, understand mental states, and recognize communicative intentions. Consequently, research on ToM in children with autism aims to enhance their social interaction skills. Kimhi (2014) suggests that combining ToM instruction with social interaction training may improve skill acquisition and generalization. This study focused solely on ToM without exploring its integration with social interaction competencies. Additionally, while teaching objectives and contextual generalization were addressed, real-life ToM assessments in home, school, and community settings were lacking. Future research should link ToM with social interaction skills, ideally in natural scenarios, to help children with autism apply learned knowledge and improve social interaction post-intervention.

Third, this study focused on basic, first-order, and first-order false beliefs in ToM, aiming to enhance children’s role-replacement competence (Wimmer & Perner, 1983). However, the research design on false beliefs has three limitations: (1) it only addresses first-order false beliefs, omitting advanced second-order false beliefs; (2) it covers only one form of first-order false beliefs (shifting position) and excludes shifting contents; and (3) it tests scenarios where subjects do not see changes, neglecting scenarios where they do. Future research should incorporate second-order false beliefs, both forms of first-order false beliefs (shifting position and contents), and scenarios where subjects see and do not see changes to better assess advanced ToM abilities.

Finally, among the intervention studies on ToM for children with ASD, some studies used computer-assisted instruction alone (Swettenham, 1996; Rice et al., 2015; Balderaz & Mehta, 2016), and there have been studies that validate the effectiveness of the thought-bubble strategy alone (Wellman et al., 2002; Zhang et al., 2016), but it is not clear which of the three teaching approaches is more effective. Future studies should compare the effectiveness of these three intervention methods or conduct a comparative study of the three methods with other methods. Furthermore, the choice of a blond-haired boy as the protagonist in the first-order false-belief task may have unintentionally attracted the attention of children with ASD, potentially affecting their performance. This indicates that future research should meticulously consider the cultural relevance of the experimental materials used.

### 4.2. Educational Implications

This study demonstrates from a practical perspective that CAI can be combined with the thought-bubble strategy to improve the ToM skills of children with ASD. When teaching ToM skills to children with ASD, teachers can use CAI combined with the thought-bubble strategy to carry out teaching activities. In terms of teaching methods, this provides new teaching ideas for the development of ToM skills in children with ASD. CAI represents a modern approach to teaching and learning. Educators can continue to explore and experiment with new teaching methods, such as virtual reality, artificial intelligence, and other technological tools, to provide children with autism with richer and more vivid learning experiences. In terms of teaching effectiveness, the ToM ability of children with ASD can be improved through careful teaching activity design. Although there are relatively large differences in the cognition and behavior of children with ASD, educators can improve their ability level by formulating appropriate teaching objectives and selecting effective teaching strategies based on the individual differences and teaching needs of the children.

## 5. Conclusions

This study investigates the effects of CAI combined with the thought-bubble strategy on the ToM abilities of children with ASD in terms of immediate, maintenance, and generalization effects. The data from this study validates the effectiveness of CAI combined with the thought-bubble strategy on the ToM abilities of children with ASD. The immediate effect of the intervention can be analyzed in terms of the last data point of the baseline session and the first data point of the maintenance session. The maintenance effect of the intervention can be analyzed from the mean of the intervention period and the maintenance period. The generalization effect of the intervention can be analyzed from the percentage of scores on the generalization data point. The analysis of the ToM test results indicated that both children’s ToM abilities improved to varying degrees before and after the intervention.

## Figures and Tables

**Table 1:** Operational definitions of dependent variables.

Target Skill	Assessment Objective	Teaching Material	Test Material (Example)	Generalization Material (Example)
Basic belief: Expressing “I feel/think”	The student is asked to talk about what they think or believe about one activity.	SD1: The apple tastes sweet. What do you think? R1: I think… I think the apple feels hard/I think the apple looks round/I think the apple tastes sour. (2 points)	It’s very hot. I want to turn on the fan. What about you?	The teacher has placed many toys on the table. SD: There are many toys here! I want to play with puzzles. What about you?
First-order belief	Child can say where the character will go and why.	After Xiao Hong got home, she found her umbrella missing. She thinks it could be on the bus or at school, but she believes her umbrella is probably at school. SD1: Where will Xiao Hong look for her umbrella? R1: The school. (thought-bubble strategy) SD2: Why will Xiao Hong look for her umbrella on the bus/at school? R2: Because Xiao Hong believes that the umbrella is at school.	Xiao Ming wants to eat an apple. The apple is either on the table or in the refrigerator, but he believes the apple is on the dining table. SD1: Where will Xiao Ming get the apple? R1: On the dining table. (1 point) SD2: Why will Xiao Ming look for the apple on the dining table/in the refrigerator? R2: Because Xiao Ming thought the apple was on the dining table. (1 point)	The teacher wants to play a video for Tu/Xuan, but can’t find the phone. The teacher thinks it might be in the backpack or cabinet, but believes the phone is in the backpack. SD1: Where will the teacher look for the phone? R1: In the backpack. (1 point) SD2: Why will the teacher look for the phone in the backpack/in the cabinet? R2: Because the teacher thinks the cell phone is in the backpack. (1 point)
First-order false belief	The student should show the thought as it happens and the reason for their thinking and actions.	Xiao Fang finished school, went home, put his schoolbag on the sofa, and went out to play. While Xiao Fang was out, his mother saw the schoolbag and moved it to Xiao Fang’s bedroom. Now, Xiao Fang has returned and wants to do his homework. SD1: Where will Xiao Fang look for his schoolbag first? R1: The sofa. (thought-bubble strategy) SD2: Why does Xiao Fang think his schoolbag is on the sofa/in the bedroom? R2: Because Xiao Fang doesn’t know that his mom took his school bag to the bedroom.	Xiao Hua put the chocolates in a basket and then left. While Xiao Hua was away, Xiao Hong came and moved the chocolates into a nearby box. Now, Xiao Hua wants to eat the chocolates. SD1: Where will Xiao Hua first look for the chocolates? R1: Inside the basket. (1 point) SD2: Why does Xiao Hua believe the chocolates are in the basket/the box? R2: Because Xiao Hua does not know that Xiao Hong put the chocolates into the box. (1 point)	Before class, Tu/Xuan put her schoolbag on the table at the doorway. After the teacher entered the classroom, she put the schoolbag into the closet. SD1: After class, where will Tu/Xuan look for her schoolbag first? R1: On the table. (1 point) SD2: Why does Tu/Xuan expect her schoolbag to be on the table/in the closet? R2: Because Tu/Xuan does not know that the teacher put the schoolbag in the cabinet.

## Data Availability

The raw data supporting the conclusions of this article will be made available by the authors upon request.

## Supplementary Materials

The following supporting information can be downloaded at: https://www.mdpi.com/article/10.3390/bs16081350/s1, Table S1: Three-Objective Teaching and Assessment Materials.
