# Supplementary material for: Enhancing Theory of Mind in Children with Autism Through Computer-Assisted Instruction and the Thought-Bubble Strategy
Source: Behav Sci (Basel). 2026 Aug 6;16(8):1350. doi: 10.3390/bs16081350 (PMC13509499; doi:10.3390/bs16081350)
Supplement: Supplementary file 1 [file behavsci-16-01350-s001.zip › behavsci-4432236-supplementary.pdf]

Table S1: Three-Objective Teaching and Assessment Materials

**Objectives**	**Types of materials**	**Content**	**Scoring criteria**
I feel/I think	Teaching materials	SD: The apple tastes sweet. What do you think?<br>R: I think... I think the apple feels hard. / I think the apple looks round. / I think the apple tastes sour. (Thought-bubble Strategy)	
	Assessment materials	1. SD: It's very hot. I want to turn on the fan. What about you?<br>R: I want to...<br>2.SD:On Sunday, I want to play in the park. What about you?<br>R: I want to...<br>3.SD: At the supermarket, I want to buy a watermelon. What about you?<br>R:I want to ......<br>4. Playing games makes me happy. What do you think?<br>R:I think ......<br>5. SD: I think flying kites is fun. What do you think?<br>R:I think	1. If the child answers "I wan" to a question about feeling cool, any appropriate cooling activity counts. (2 points)<br>2. Answering "I want" to a leisure activity like going to an amusement park is acceptable. (2 points)<br>3. Answering "I want" to buying a specific supermarket item, such as an ice cream bar, is acceptable. (2 points)<br>4. Describing that playing games or similar activities brings relaxation or joy is acceptable. (2 points)<br>5. If a child says flying kites is difficult, expressing related feelings like happiness or relaxation using "I think" is acceptable. (2 points)
	Generalization materials	Scenario: The teacher has placed many toys on the table.<br>SD: There are many toys here! I want to play with puzzles. what about you?<br>R: I want to...	I want to play with Lego. Any response using "I want" is acceptable. (2 points)
		Scenario: The teacher has placed many picture books on the table.<br>SD: There are many picture books here. I want to read this one. what about you?<br>R: I want to...	I want to read this book. Any response using "I want" is acceptable. (2 points)

	Teaching materials	After Xiao Hong got home, she found her umbrella missing. She thinks it could be on the bus or at school, but she believes her umbrella is probably at school.<br>SD1:Where will Xiao Hong look for her umbrella?<br>SD2: Why will Xiao Hong look for her umbrella on the bus /at school?	R1: The school.(Thought-bubble Strategy)<br>R2: Because Xiao Hong believes that the umbrella is at school.
First-order Beliefs	Assessment materials	1. Xiao Ming wants to eat an apple. He thinks it could be on the dining table or in the refrigerator, but he believes the apple is on the dining table.<br>SD1:Where will Xiao Ming get the apple?<br>SD2: Why will Xiao Ming look for the apple on the dining table /in the refrigerator?	R1:On the dining table. (1 point)<br>R2: Because Ming thought the apples were on the dining table. (1 point)
		2.Xiao Zhang wants to play a game with Xiao Bai, who he thinks might be in the classroom or the toilet. Xiao Zhang believes Xiao Bai is in the toilet.<br>SD1:Where will Xiao Zhang look for Xiao Bai?<br>SD2: Why will Xiao Zhang look for Xiao Bai in the toilet /in the classroom?	R1:Toilet. (1 point)<br>R2: Because Xiao Zhang thinks Xiao Bai is in the toilet. (1 point)
		3. Xiao Liu wants to buy a birthday gift. His mother might like cake or flowers, but he believes she prefers cake.<br>SD1: What will Xiao Liu buy for his mother?<br>SD2: Why will Xiao Liu buy a cake /flowers for his mother?	R1:Cake. (1 point)<br>R2: Because Xiao Liu thinks his mom prefers cake. (1 point)
		4.Xiao Hei wants to drink water. He thinks the water cup may be in the kitchen or bedroom, but he believes it's in the kitchen.<br>SD1:Where will Xiao Hei look for the water cup?<br>SD2: Why will Xiao Hei look for the water cup in the kitchen /in the bedroom?	R1:Kitchen. (1 point)<br>R2: Because Xiao Hei thinks the water cup is in the kitchen. (1 point)
		5.Dou Dou wants to bring snacks to Xiao Yu. Dou Dou thinks Xiao Yu might like potato chips or biscuits, but since he believes she prefers potato chips.<br>SD1:What will Dou Dou bring Xiao Yu?<br>SD2: Why will Dou Dou bring potato chips /biscuits to Xiao Yu?	R1:potato chips. (1 point)<br>R2: Because Dou Dou thinks Xiao Yu likes potato chips. (1 point)
	Generalization materials	The teacher wants to play a video for Tu/Xuan, but can't find the phone. The teacher thinks it might be in the backpack or cabinet, but believes the phone is in the backpack.<br>SD1:Where will the teacher look for the phone?	R1:In the backpack. (1 point)

		SD2: Why will the teacher look for the phone in the backpack /in the cabinet?	R2:Because the teacher thinks the cell phone is in the backpack. (1 point)
		The teacher wants to draw with Tu/Xuan but can't find the colored pencils. The teacher thinks the colored pencils might be in another classroom's cabinet or the school bag, but believes the pencils are in the neighboring classroom's cabinet.<br>SD1:Where will the teacher look for the colored pencils?<br>SD2: Why will the teacher look for the colored pencils in the cabinet /in the school bag?	R1:Home. (1 point)<br>R2:Because the teacher thinks the colored pencils are at home. (1 point)
	Teaching materials	Xiao Fang finished school, went home, put his schoolbag on the sofa, and went out to play. While Xiao Fang was out, his mother saw the schoolbag and moved it to Xiao Fang's bedroom. Now, Xiao Fang has returned and wants to do his homework.<br>SD1: Where will Xiao Fang look for his schoolbag first?<br>SD2: Why does Xiao Fang think his schoolbag is on the sofa /in the bedroom?	R1: The sofa. (Thought-bubble Strategy)<br>R2: Because Xiao Fang doesn't know that his mom took his school bag to the bedroom.
First -order False Beliefs	Assessment materials	1.Little Mouse put the watermelon in the refrigerator and then went to play with toys. While Little Mouse was playing, the giraffe opened the refrigerator, took out the watermelon, and put it into a bag. Now, Little Mouse wants to eat the watermelon.<br>SD1: Where will Little Mouse look for the watermelon first?<br>SD2: Why does Little Mouse believe the watermelon is in the refrigerator /the bag?<br>2.Xiao Hua put the chocolates in a basket and then left. While Xiao Hua was away, Xiao Hong came and moved the chocolates into a nearby box. Now, Xiao Hua wants to eat the chocolates.<br>SD1: Where will Xiao Hua first look for the chocolates?<br>SD2: Why does Xiao Hua believe the chocolates are in the basket /the box?<br>3. Xiao Qiang put his homework on the desk and went to sleep. While he was asleep, his mother saw the homework and put it into his schoolbag. The next morning, Xiao Qiang wakes up to look for his homework.<br>SD1: Where will Xiao Qiang look first for his homework?<br>SD2: Why does Xiao Qiang expect his homework to be on the desk /in his	R1: The refrigerator. (1 point)<br>R2: Because Little Mouse does not know that the giraffe put the watermelon into the bag. (1 point)<br>R1: Inside the basket. (1 point)<br>R2: Because Xiao Hua does not know that Xiao Hong put the chocolates into the box. (1 point)<br>R1: On the desk. (1 point)<br>R2: Because Xiao Qiang does not know that his mother put the homework into the schoolbag. (1

	schoolbag?	point)
	4.Xiao Xin gave the toys to his father and then went out. While Xiao Xin was away, his father gave the toys to his mother. Now that Xiao Xin has returned home, he wants to play with the toys.<br>SD1: Who will Xiao Xin ask for the toys first?<br>SD2: Why does Xiao Xin believe his father /mother has the toys?	R1: Dad. (1 point)<br>R2: Because Xiao Xin does not know that his dad gave the toys to his mother. (1 point)
	5.Dad came home from work, put his phone on the living room table, and went to take a shower. While Dad was showering, Xiao Huang saw his father's phone and took it to his bedroom to play. Now, Dad has finished his shower.<br>SD1: Where will Dad look for his phone first?<br>SD2: Why does Dad think his phone is on the table /in the bedroom?	R1: On the living room table. (1 point)<br>R2: Because Dad does not know that Xiao Huang took the phone to the bedroom.
Generalization materials	Mom took Tu/Xuan to school, placed the water cup on the table, and waited outside the classroom for Tu/Xuan to finish class. Before class, the teacher put the water cup on the shelf. After class, Mom came to pick up Tu/Xuan from school.<br>SD1: Where will Mom first look for the water cup?<br>SD2: Why will Tu/Xuan expect the water cup to be on the table /on the shelf?	R1: On the table. (1 point)<br>R2: Because Mom does not know that the teacher placed the water cup on the shelf.
	Before class, Tu/Xuan put her schoolbag on the table at the doorway. After the teacher entered the classroom, she put the schoolbag into the closet.<br>SD1: After class, where will Tu/Xuan look for her schoolbag first?<br>SD2: Why does Tu/Xuan expect her schoolbag to be on the table /in the closet?	R1: On the table. (1 point)<br>R2: Because Tu/Xuan does not know that the teacher put the schoolbag in the cabinet.
